# Identifying the signature of prospective motor control in children with autism

**DOI:** 10.1038/s41598-021-82374-2

**Published:** 2021-02-04

**Authors:** Andrea Cavallo, Luca Romeo, Caterina Ansuini, Francesca Battaglia, Lino Nobili, Massimiliano Pontil, Stefano Panzeri, Cristina Becchio

**Affiliations:** 1 Cognition, Motion and Neuroscience Laboratory, Center for Human Technologies, Istituto Italiano di Tecnologia, Genoa, Italy; 2 Department of Psychology, Università degli Studi di Torino, Turin, Italy; 3 Department of Information Engineering, Università Politecnica delle Marche, Ancona, Italy; 4 Computational Statistics and Machine Learning Laboratory, Center for Human Technologies, Istituto Italiano di Tecnologia, Genoa, Italy; 5 Child Neuropsychiatric Unit, IRCCS Istituto G. Gaslini, Genoa, Italy; 6 DINOGMI Department of Neurosciences, Rehabilitation, Ophthalmology, Genetics and Maternal and Children’s Sciences, Università degli Studi di Genova, Genoa, Italy; 7 Neural Computational Laboratory, Center for Human Technologies, Istituto Italiano di Tecnologia, Genoa, Italy

**Keywords:** Motor control, Sensorimotor processing, Autism spectrum disorders

## Abstract

Failure to develop prospective motor control has been proposed to be a core phenotypic marker of autism spectrum disorders (ASD). However, whether genuine differences in prospective motor control permit discriminating between ASD and non-ASD profiles over and above individual differences in motor output remains unclear. Here, we combined high precision measures of hand movement kinematics and rigorous machine learning analyses to determine the true power of prospective movement data to differentiate children with autism and typically developing children. Our results show that while movement is unique to each individual, variations in the kinematic patterning of sequential grasping movements genuinely differentiate children with autism from typically developing children. These findings provide quantitative evidence for a prospective motor control impairment in autism and indicate the potential to draw inferences about autism on the basis of movement kinematics.

## Introduction

Much autism research has focused on the social, communication and cognitive difficulties associated with the condition. However, recent years have seen an increasing interest in the motor side of autism spectrum disorders (ASD)^1^, with several researchers going as far as proposing that impairments in the prospective control of movements^2,3^ may be predictive of ASD and may even underlie some of the core features of ASD^4^. Despite this enthusiasm, relatively little quantitative information is available about prospective movement alterations in ASD and questions remain regarding whether (and to what extent) kinematic patterning permit differentiating ASD and non-ASD profiles over and above individual differences in motor output^5^.

Recently, researchers have started to address these questions by applying machine learning as a tool for multivariate analysis with the goal of finding inherent patterns in kinematic data. In a typical application, a machine learning classifier is trained to distinguish ASD and non-ASD movement patterns on one part of a data set. Then, the classifier is tested on the remaining data. This results in a certain fraction of accurate classifications. If the classification accuracy lies significantly above the level expected by chance (e.g., 0.50), it can be concluded that a difference between classes exists. Studies applying this approach to prospective movement data suggest that patterns related to ASD can be classified with near-perfect accuracy^6,7^. The caveats inherent to applications of machine learning methods for identifying group differences in movement data, however, are rarely considered^8^.

A first caveat relates to confound variables, that is, variables that in the selected sample have an association with diagnosis group, but are uninteresting from a clinical perspective^9^. Consider, for example, movement variations related to age. In a sample in which ASD children, say, are younger than typically developing (TD) children^7^, the classifier is not only learning movement features discriminating ASD and TD movement, but also age-related features. This can lead to overestimate the information that is computationally available to differentiate ASD and TD movements.

A second caveat relates to individual variations in motor outputs. Individuals show variations in motor outputs that are both consistent within a given individual and differ from one individual to another^10–13^. When repeated measures from the same individual are randomly assigned to training and testing as in common machine learning approaches using record-wise cross-validation^6^, algorithms can learn the association between individuals’ movement signature and their diagnostic label. This can lead to identity-confounded predictions, where the easier task of identity classification de facto circumvents and replaces the harder task of diagnostic classification^14^.

Both these caveats are addressed in the present study. In a sample of cases accurately matched on age, gender, full-scale IQ, and stature, we use machine learning methods as a tool to determine the true power of prospective movement data to differentiate ASD and TD group profiles and predict the group of a previously unseen individual. We do so by contrasting record-wise data splits, where repeated measurements of each individual are assigned to both the training set and the test set, with subject-wise data splits, where any source of identity confound is neutralized by the assignment of all measurements of each subject to either the training set or the test set^14^ (see “Methods” section for details). Our results show that although movement is unique to each individual, differences in prospective motor control genuinely differentiate ASD from TD movement data.

## Results

**Figure 1:**
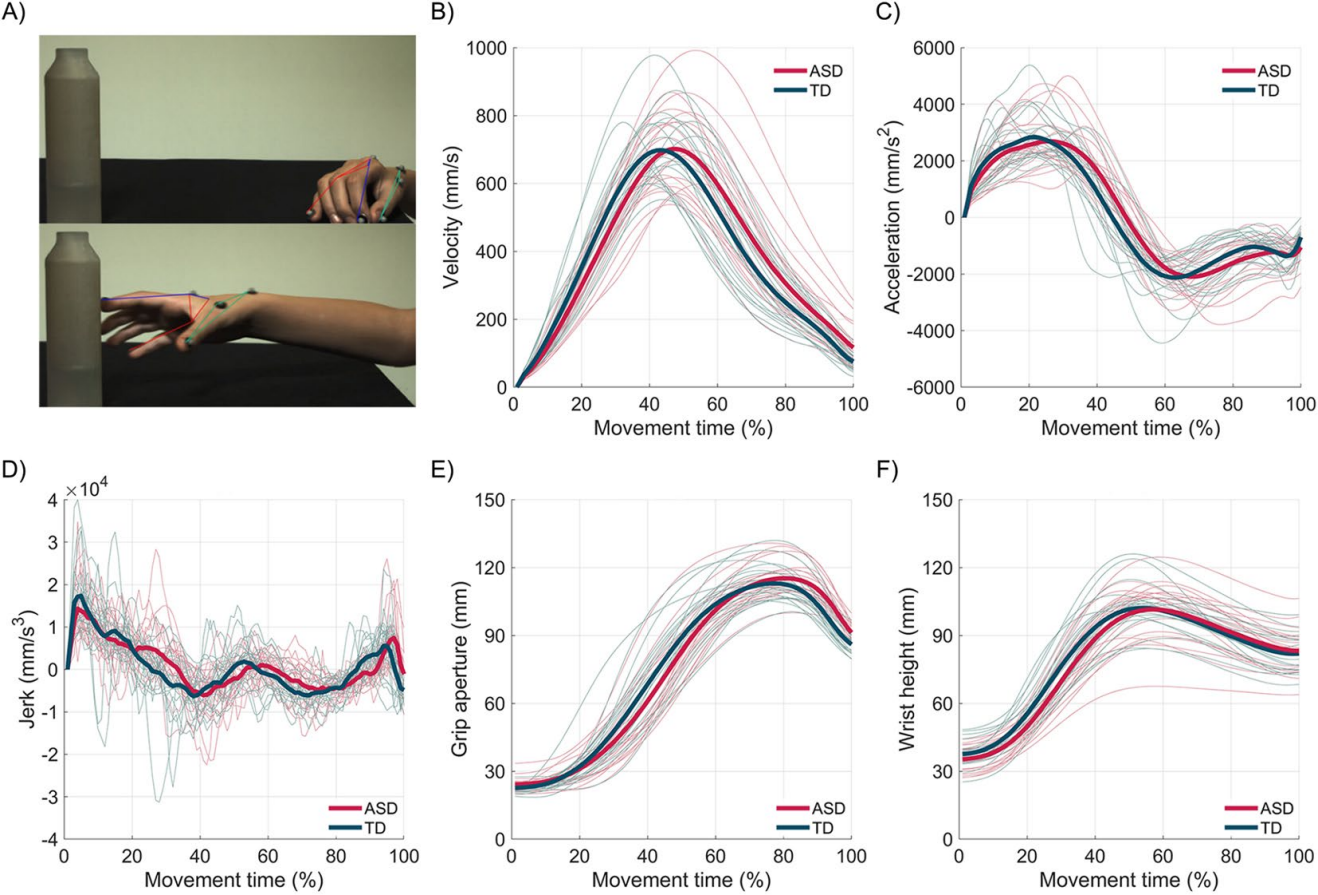
(**A**) Image sequence of a reach-to-grasp movement with hand model overlay. (**B–F**) Kinematic profiles of reach-to-grasp movements performed with three prospective intentions (to place, to pour, to pass). Wrist velocity (**B**), wrist acceleration (**C**), wrist jerk (**D**), grip aperture (**E**) and wrist height (**F**) were extracted. Thick lines represent group average (red = ASD group, green = TD group). Thin lines represent trial-average of individual participants.

Data for this study consisted of 1600 movements recorded from 20 ASD and 20 TD children reaching towards and grasping a bottle with one of three prospective intentions: place the bottle into a box (grasp-to-place), pour some water (grasp-to-pour) or pass the bottle to another person (grasp-to-pass; see “Methods” section). Visually, movement traces showed high variability across individuals (Fig. 1B–F) and trials (Supplementary Fig. S1), with variations across individuals greatly exceeding variations between groups (ASD, TD) in all extracted variables.

### Intention classification

To quantify prospective modulation, we first attempted to classify intention, operationalized as onward action (place, pour, pass), from reach-to-grasp parameters, separately for TD and ASD data. Cross-validation using record-wise splits revealed that, in both groups, kinematics predicted intention above chance (TD mean ± SEM = 0.64 ± 0.02; *p* < 0.001, permutation test; ASD mean ± SEM = 0.57 ± 0.02; *p* < 0.001, permutation test). In line with^15^, prospective modulation was less pronounced in ASD movement profiles compared to TD movement profiles as indicated by a lower classification accuracy (*p* = 0.039, sign test).

### Group classification

**Figure 2:**
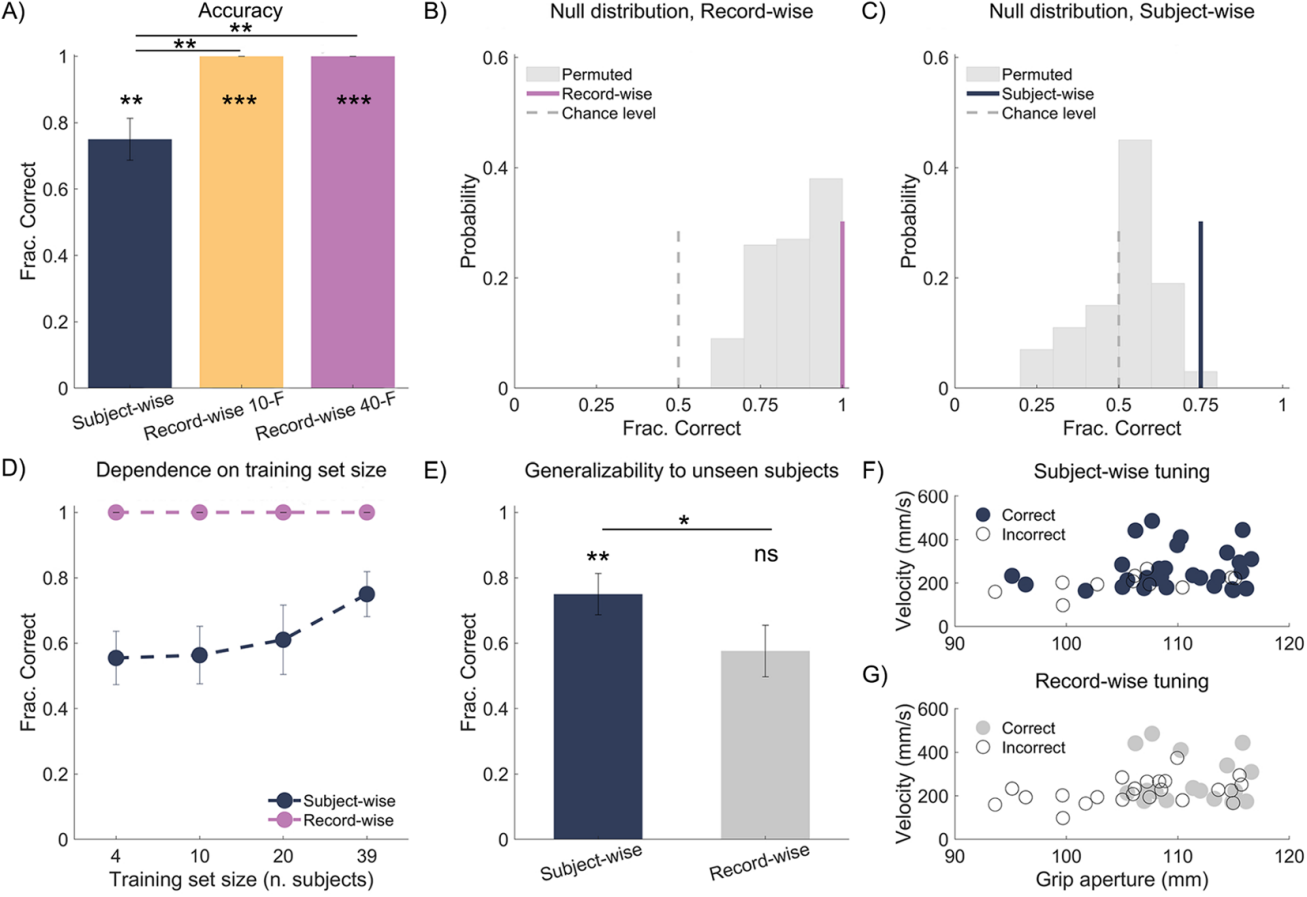
(**A**) Classification accuracy of group (ASD, TD) using subject-wise, record-wise 10 folds cross-validation and record-wise 40 folds cross-validation. Histograms represent mean ± SEM. (**B**,**C**) Permutation null distribution generated by splitting data in a record-wise fashion (**B**) or subject-wise fashion (C). The permutation null distribution is represented by the grey histograms. The purple line in (**B**) represents the observed record-wise accuracy. The dark blue line in (**C**) represents the observed subject-wise accuracy. The dashed grey line indicates the 0.5 chance level. (**D**) Group classification accuracy as a function of sample size computed using record-wise and subject-wise cross-validation. (**E**) Group classification accuracy of unseen subjects obtained using subject-wise hyper-parameter tuning and record-wise hyper-parameter tuning. (**F**,**G**) Movement data colored according to group classification of one exemplar unseen subject with ASD using subject-wise hyper-parameter tuning (**F**) and record-wise hyper-parameter tuning (**G**). Data are visualized as a scatterplot of velocity at 80% of movement duration and grip aperture at 90% of movement durations.

After establishing reduced prospective modulation in ASD compared to TD data, we next sought to determine the power of prospective movement data to differentiate ASD and TD movement profiles. Analysis of simulated^14^ and empirical data^16^ suggest that classifiers trained and evaluated using record-wise data splits can pick confounding relationship between identity and group and so produce inflated accuracies. To address this concern in our data, we contrasted record-wise data splits, where repeated measurements from the same individual are assigned to both the training set and the test set, with subject-wise data splits, where measures of each subject are assigned to either the training set or test set, neutralizing any potential identity confounding^14^. Classifiers trained and evaluated using record-wise splits achieved perfect accuracy in discriminating ASD versus TD (accuracy mean ± SEM = 1.00 ± 0.00; sensitivity mean ± SEM = 1.00 ± 0.00; specificity mean ± SEM = 1.00 ± 0.00). As a control, we repeated record-wise splitting over 40 folds and found again that all cases were correctly classified (accuracy mean ± SEM = 1.00 ± 0.00; sensitivity mean ± SEM = 1.00 ± 0.00; specificity mean ± SEM = 1.00 ± 0.00). Classification accuracies of classifiers trained with subject-wise data splits (accuracy mean ± SEM = 0.75 ± 0.06; sensitivity mean ± SEM = 0.75 ± 0.09; specificity mean ± SEM = 0.75 ± 0.08) were significantly lower (*p* = 0.002, sign test), although still significantly above chance (*p* = 0.010, permutation test) (Fig. 2A). We obtained similar results using SVM-LASSO and RF (Supplementary Fig. S2).

To evaluate the influence of the training set size on classification accuracy, we considered four sets of classifiers in which the training set was composed of 4, 10, 20 or 39 subjects. The accuracy of classifiers built using record-wise splits was already perfect with 4 subjects and remained so through the four sets (Fig. 2D), raising the concern that record-wise classifiers fit to the idiosyncratic characteristics of the training sample^17,18^. In contrast, the accuracy of classifiers built using subject-wise data splits was at chance with 4 and 10 subjects, increased to 0.61 with 20 subjects and continued to increase with 39 subjects, suggesting that subject-wise classifiers learned group information from new examples.

### Quantification of identity confounding

The above results are compatible with the notion that identity confounding artificially inflates classification accuracies using record-wise data splits. To confirm the presence of identity information, as a first step, we tested whether group could be identified based on identity. In this analysis, we trained classifiers with record-wise splits to predict the identity of participants rather than the group. Consistent with the possibility that the power of record-wise classifiers is based on identity information, this analysis yielded perfect identity classification (accuracy mean ± SEM = 1.00 ± 0.00; sensitivity mean ± SEM = 1.00 ± 0.00; specificity mean ± SEM = 1.00 ± 0.00).

To more directly test whether record-wise classifiers learn identity information rather than group information, we next randomly permuted the diagnostic labels of each subject as a block, so that all records of a given subject were assigned either ASD or TD during the permutation process^16,19^. The motivation for this relabelling scheme is that it preserves the confounding association between group and identity, while breaking the relationship between movement data and group. Because group information is removed, permutation null distributions located on average above the 0.5 chance level indicate identity confounding^19^. Conforming an identity confounding, the null distribution of classification accuracies obtained from record-wise data splits had all data distributed above the 0.5 chance level (Fig. 2B). To assess whether classifiers were learning group information in addition to identity information, we compared the location of the record-wise classification accuracy computed using the original (unpermuted) data relative to the permuted data. Only if classification accuracy computed using the original data is higher than that of permuted data, it can be concluded that the classifier is learning group information. Record-wise accuracy computed using the original data did not differ from the permutation null distribution (*p* = 0.170). In contrast to record-wise data splits, the null distribution generated by splitting data in a subject-wise fashion was located around 0.5 (Fig. 2C). Also, subject-wise accuracy computed using the original data was significantly higher than the adjusted null distribution (*p* = 0.01, permutation test, Fig. 1D). Overall, these results support the conclusion that classifiers trained subject-wise, but not classifiers trained record-wise, learned differences between ASD and TD movements.

### Generalizability to unseen cases

If a classifier is learning group information, its predictive power should generalize to unseen cases. In contrast, if a classifier is learning identity information, its accuracy should drop on unseen cases. Based on these predictions, we compared the ability of classifiers trained record-wise and subject-wise to predict group for unseen cases. To quantify the ability of classifiers trained record-wise to predict group for an unseen individual, we used a hybrid cross-validation procedure^20^ in which hyper-parameters were tuned record-wise in the inner loop (model validation), whilst model performance was recursively tested subject-wise in the outer loop (model testing) on a new individual. Under this procedure, classifiers tuned record-wise (accuracy mean ± SEM = 0.58 ± 0.08; sensitivity mean ± SEM = 0.55 ± 0.12; specificity mean ± SEM = 0.60 ± 0.10) performed at chance level (*p* = 0.110, permutation test), significantly worse than classifiers tuned subject-wise at classifying unseen cases (*p* = 0.023, sign test; see Fig. 2E). Figure 2F,G provide an example of classification of the movements of a new, unseen ASD participant using subject-wise (Fig. 2F) and record-wise (Fig. 2G) tuning. Data are visualized in a reduced kinematic space spanning only two kinematic features, velocity at 80% of movement duration and grip aperture at 90% of movement duration. Compared to the number of movements assigned to the correct group (ASD) using subject-wise data splits (30 out of 40; Fig. 2F), the number of correctly classified movements substantially decreases using record-wise data splits (17 out of 40; Fig. 2G). As a result, the participant’s group (ASD) is correctly classified using subject-wise tuning, but not record-wise tuning.

### Intention-specific contribution to group classification

Subject-wise data splits indicate that prospective movement data contain group discriminating information that generalizes to unseen cases. To examine how such information is encoded in specific intention profiles, we attempted to predict group separately from kinematic parameters of grasp-to-place, grasp-to-pour and grasp-to-pass movements. Supplementary Fig. S3 visualizes individual movement profiles graphed by intention. Separating intentions revealed that group differences were predominantly located in grasp-to-pass and grasp-to-pour movements. Characteristically, ASD grasp-to-pass showed later temporal setting of peak wrist velocity, peak wrist acceleration and peak wrist deceleration compared to TD grasp-to-pass. ASD grasp-to-pour also showed later peak wrist velocity and peak wrist deceleration compared to TD grasp-to-pour. Consistent with the visual impression gained from Supplementary Fig. S3, classification of group was most accurate from grasp-to-pass movements (accuracy mean ± SEM = 0.83 ± 0.05; sensitivity mean ± SEM = 0.80 ± 0.08; specificity mean ± SEM = 0.85 ± 0.08), followed by grasp-to-pour movements (accuracy mean ± SEM = 0.68 ± 0.07; sensitivity mean ± SEM = 0.60 ± 0.11; specificity mean ± SEM = 0.75 ± 0.09) and grasp-to-place movements (accuracy mean ± SEM = 0.60 ± 0.07; sensitivity mean ± SEM = 0.65 ± 0.12; specificity = 0.55 ± 0.12). Classification accuracy was above chance for grasp-to-pass (*p* < 0.001) and grasp-to-pour (*p* = 0.02) movements, but not for grasp-to-place movements (*p* = 0.380). This indicates that grasp-to-pass and grasp-to-pour movements, but not grasp-to-place movements, individually contributed group discriminating information.

## Discussion

Developments in machine learning and the increasing availability of motion tracking devices have brought movement to the forefront of autism research, diagnosis and treatment^4,21–23^. However, questions remain regarding whether and to what extent kinematic patterning permit differentiating ASD and non-ASD movement profiles^5^.

Here, we assessed the potential of multivariate prospective motor control data to discriminate movements performed by ASD children from TD children in a sample of cases matched on age, gender, full-scale IQ, and stature. By comparing record-wise and subject-wise cross-validation approaches in combination with different machine learning classifiers, we demonstrate that record-wise data splitting leads to an optimistic bias in accuracy. We show that such bias is inadvertently introduced in record-wise computations by individual movement characteristics. Individuals performing prospective grasps exhibit repeatable aspects of their movement signature across trials^10^. When data collected from the same individual are assigned to both the training and the test sets (as in record-wise cross-validation), models learn the idiosyncratic movement signature of individual participants, rather than the signature that separates ASD and TD movements. The upshot is that the apparent perfect classification accuracy achieved by classifiers trained record-wise drops to chance level on unseen cases.

Whit subject-wise cross-validation, the accuracy of prospective movement data in predicting group decreases—the best classifier achieving 0.75 classification accuracy—but remains statistically significant. Because data from each individual are assigned to either the training or the test set, subject-wise accuracies represent the true accuracy of prospective movement data in predicting group.

Separating data into intention-specific profiles revealed that group information is predominantly encoded in grasp-to-pass prospective movements. Unlike individual sequential actions such as grasp-to-place, passing an object requires the coordination—in space and time—between two partners^24–26^. One interpretation of this result is that ASD atypical grasp-to-pass profiles relate to the social nature of the task. However, another possibility is that atypical grasp-to-pass profiles relate to the semi-predictable nature of the passing task^27^. During passing, each partner may have a basic internal model regarding the evolution of the passing movement. However, given that control by one partner cannot be fully predicted by the other partner, there will necessarily be unpredictable aspects to the task^28^. We propose that the later temporal setting of key landmarks in ASD grasp-to-pass movements may reflect reduced motor anticipation under conditions of uncertainty. A similar explanation may hold for grasp-to-pour movements, which have been shown to involve demanding probabilistic inferences about fluid dynamics over short time scales^29^. This account makes the prediction, testable in future studies, that ASD and TD movement profiles become more distinct under conditions of increased volatility^30^.

In our study, we chose to measure a homogeneous sample, carefully selected based on a specific set of inclusion and exclusion criteria to rule out a number of potential confounders. However, models based on homogeneous samples are less likely to generalize to real-life clinical settings, where patient groups are highly heterogeneous^31^. To dissect the heterogeneity of ASD, future studies should compare prospective motor control in ASD children with and without intellectual disability, and in ASD children classified as having comorbid attention and/or motor impairments. Such knowledge could help identifying ASD subtypes most likely to respond to specific interventions and developing personalized treatments.

Future research should also evaluate the specificity and sensitivity of ASD movement profiles relative to other neurodevelopmental disorders, such as attention deficit hyperactivity disorder and developmental coordination disorder. Testing specificity over multiple alternatives is important to establish whether prospective motor impairments in ASD could represent a putative endophenotype with potential to elucidate pathophysiology of ASD and facilitate early diagnosis^5^.

Finally, it will be important for future studies to assess the potential of prospective data to identify ASD in larger samples. In this regard, the steady increase in prediction accuracy observed using subject-wise data splits as a function of the size of training set—far from plateauing with 20 subjects per class—shows promise, suggesting that prediction of unseen cases could be improved when training models on larger samples.

## Conclusions

The study reports on a promising advance in objectively characterizing movement kinematics in ASD. Our approach enabled us to isolate the information that genuinely differentiates ASD and TD movement profiles and to quantify the identity confounding inherent to record-wise cross-validation. Combined, our findings invite a reassessment of recent conclusions about the diagnostic power of prospective movement data. The finding that prospective movement data contain identity information, but also genuine group information highlights the utility of machine learning methods as a tool for developing robust single-subject predictor to accurately differentiate autism and/or subtypes of autism.

## Methods

### Participants

We report results from 20 ASD children (18 males) without accompanying intellectual impairment and 20 typically developing children (16 males). Groups were matched for age (TD mean ± SD = 9.5 ± 1.5 years, months; ASD mean ± SD = 9.8 ± 1.5 years, months; t_38_ =−0.665, *p* = 0.510), stature (TD mean ± SD = 140.4 ± 8.1 cm; ASD mean ± SD = 137.7 ± 9.1 cm; t_38_ = 0.988, *p* = 0.329) gender, and Full Scale IQ as measured by the Wechsler Scale of Intelligence (WISC IV)^32^ (TD mean ± SD = 102.8 ± 9.4; ASD mean ± SD = 98.5 ± 11.1; t_38_ = 1.325, *p* = 0.193). Children with ASD were diagnosed according to DSM-5 criteria^33^. The Autism Diagnostic Observation Scale (ADOS-2)^34^ and Autism Diagnostic Interview-Revised (ADI-R)^35^ were administered by two experienced professionals. All children had normal or corrected-to-normal vision and were screened for exclusion criteria (pharmacological treatment, epilepsy, and any other neurological and psychiatric conditions). Both ASD and TD groups were assessed for executive functions abilities by means of the Tower of London (TOL) test^36^. This test revealed no significant differences between TD and ASD children (TD mean ± SD = 29.35 ± 3.54; ASD mean ± SD = 29.35 ± 2.80; t_38_ = 0, *p* = 0.999). All but two of the children (one in the ASD group and one in the TD group) were right-handed according to the Edinburgh Handedness Inventory^37^. Written informed consent was obtained from the parents of the children prior to participation in the experiment. The research protocol was approved by the local ethics committee (Comitato Etico Regionale Liguria) and was in accordance with the principles of the revised Helsinki Declaration (2013)^38^.

### Experimental design

Children were seated on a height-adjustable chair with their right elbow and wrist resting on a table (height = 64 cm; length = 100 cm; width = 60 cm). A plastic bottle filled with water (base diameter = 5 cm; height = 18 cm; weight = 225 g) was positioned on the table at a distance of 44 cm from children’s midline. Children were asked to reach for and grasp the bottle to complete three sequential manipulation tasks: place the bottle into a box (grasp-to-place), pour some water into a glass (grasp-to-pour), or pass the bottle to a co-actor (grasp-to-pass), who would then either place the bottle into the box or pour some water. In each trial, children were asked to perform at a natural speed after an auditory tone. Each child completed 4 blocks of 12 trials (1 grasp-to-place block, 1 grasp-to-pour block, and 2 grasp-to-pass blocks). The order of blocks was pseudo-randomized across participants. The experiment lasted about 30 min, with a 2-min pause at the end of each block. Throughout the entire experimental session, the same female experimenter (co-actor) sat at the opposite side of the table and interacted with the child.

### Kinematics recording and data processing

A near-infrared camera motion capture system equipped with six cameras (frame rate = 100 Hz; Bonita Vicon Motion Systems Ltd, Oxford, UK) was used to track and record the reach-to-grasp kinematics. The child’s right hand was outfitted with 8 retro-reflective hemispheric markers (6.5 mm in diameter) placed on the metacarpal joint and the tip of the index and the little finger, the trapezium bone of the thumb, the radial aspect of the wrist and the centre of the hand dorsum (Fig. 1A). After data collection, each trial was individually inspected for correct marker identification and then run through a 6 Hz low-pass Butterworth filter^15^. Trials in which the quality of markers reconstruction was poor were discarded from the dataset and not considered for further analyses. To equate the total number of trials between blocks and participants, the final dataset consisted of 10 trials per block per participant, for a total of 1600 movements. A custom software written in Matlab (MathWorks, Natick, MA) was used to compute wrist velocity, wrist acceleration, wrist jerk, wrist height, and grip aperture. Each variable was computed at intervals of 10% of movement duration, from reach onset to reach offset (see Supplementary Table S1).

### Classification analyses

#### Intention classification

We computed classification of onward action (place, pour, pass) from reach-to-grasp parameters based on a Support Vector Machine (SVM) model, separately for TD and ASD movement data. The SVM used Gaussian kernel (SVM-G) to compute the hyperplane that best separated grasp-to-place, grasp-to-pour and grasp-to-pass trials in each diagnosis group. To equate the number of trials across action classes, for grasp-to-pass we randomly selected 5 trials per block. For each diagnosis group, the dataset thus comprised 600 reach-to-grasp movements (20 participants × 10 trials × 3 actions). Models were trained, validated and tested using a record-wise cross-validation data split (with data split into 10 folds, each fold containing 1 movement for each intention for each participant). Classification accuracy was computed as the fraction of correctly classified trials for each cross-validation iteration.

#### Group classification

For this analysis, we used all 1600 reach-to-grasp movements (40 participants × 10 trials × 4 blocks). We computed group (ASD, TD) from reach-to-grasp parameters based on a SVM-G model. For comparison, we also computed group based on SVM regularized with least absolute shrinkage and selection operator (SVM-LASSO) and Random Forest (RF). Models were evaluated using record-wise cross-validation and subject-wise cross validation. For record-wise cross-validation, we split data into 10 (40) folds so that each fold contained 4 (1) movements from each subject. For subject-wise cross-validation, we split data by subjects such that training and test folds contained records from different subjects. In both methods, hyper-parameters were tuned recursively on all but one fold of the training set and tested on the remaining fold. For each subject, group was determined based on the averaged posterior scores of the movements of that subject. Classification accuracy was computed as the fraction of correctly classified subjects. Sensitivity—the fraction of ASD cases correctly classified as ASD in reference to all ASD cases—and specificity—the fraction of TD cases correctly classified as TD in reference to all TD cases—were also computed as measures of classifier performance.

#### Intention-specific contribution to group classification

To evaluate the diagnostic power of specific action sequences, we also computed group separately from grasp-to-place, grasp-to-pour and grasp-to-pass movements based on three separate SVM-G models. Models were validated and tested using subject-wise cross validation.

### Statistics of model performance

In all analyses, we assessed model performance using classification accuracy. We evaluated the significance of classification accuracy against chance with permutation statistics. The chance-level null-hypothesis distribution of these statistics was created by computing classification accuracy after randomly permuting the trial label of each individual trial for the record-wise calculations and the trial label of all trials of a given subject as block for the subject-wise calculations (100 random permutations). We evaluated the significance of differences in how accurately different models classified intentions (intention classification) or group (group classification) using two-tailed paired-sample sign tests. We used non-parametric tests because classification accuracies do not follow a Gaussian distribution. Standard error of the mean (SEM) were computed by bootstrapping for reporting, but their value was not used in the computation of the non-parametric statistics of model performance.

*Notes* In all figures, * indicates *p* < 0.05, ** indicates *p* ≤ 0.01, *** indicates *p* ≤ 0.001. Following standard notations, asterisks above bars indicate above chance significance, asterisks above connecting lines indicate comparison significance.

### Community involvement

We consulted clinical practitioners from the local hospital on how to create an enabling environment and plan experimental procedures to maximise the engagement and enjoyment of the children. Their feedback was incorporated in the study design. We shared the aggregate study results with children and their families in a 1-day workshop organized by the research team in partnership with clinical practitioners.

## Supplementary Information

Supplementary Informations.

## Data Availability

The data supporting the main findings of the current study are available from the corresponding author on reasonable request. The code supporting the main findings is based on public available tools as detailed in “Methods” section. Custom functions inputting data to toolboxes will be made available by the corresponding author upon reasonable request.

## References

[1] Sacrey L-AR, Germani T, Bryson SE, Zwaigenbaum L (2014). Reaching and grasping in autism spectrum disorder: a review of recent literature. Front. Neurol..

[2] von Hofsten C (2004). An action perspective on motor development. Trends Cogn. Sci..

[3] Rosenbaum DA, Chapman KM, Weigelt M, Weiss DJ, van der Wel R (2012). Cognition, action, and object manipulation. Psychol. Bull..

[4] Trevarthen C, Delafield-Butt JT (2013). Autism as a developmental disorder in intentional movement and affective engagement. Front. Integr. Neurosci..

[5] Hocking DR, Caeyenberghs K (2017). What is the nature of motor impairments in autism, are they diagnostically useful, and what are the implications for intervention?. Curr. Dev. Disord. Rep..

[6] Anzulewicz A, Sobota K, Delafield-Butt JT (2016). Toward the autism motor signature: gesture patterns during smart tablet gameplay identify children with autism. Sci. Rep..

[7] Crippa A (2015). Use of machine learning to identify children with autism and their motor abnormalities. J. Autism Dev. Disord..

[8] Bone D (2014). Applying machine learning to facilitate autism diagnostics: pitfalls and promises. J. Autism Dev. Disord..

[9] Rao A, Monteiro JM, Mourao-Miranda J (2017). Predictive modelling using neuroimaging data in the presence of confounds. NeuroImage.

[10] Koul A, Cavallo A, Ansuini C, Becchio C (2016). Doing it your way: how individual movement styles affect action prediction. PLoS ONE.

[11] Ting LH (2015). Neuromechanical principles underlying movement modularity and their implications for rehabilitation. Neuron.

[12] Richardson MJ, Johnston L (2005). Person recognition from dynamic events: the kinematic specification of individual identity in walking style. J. Nonverbal Behav..

[13] Patri J-F (2020). Transient disruption of the inferior parietal lobule impairs the ability to attribute intention to action. Curr. Biol..

[14] Saeb S, Lonini L, Jayaraman A, Mohr DC, Kording KP (2017). The need to approximate the use-case in clinical machine learning. Gigascience.

[15] Cavallo A (2018). Prospective motor control obeys to idiosyncratic strategies in autism. Sci. Rep..

[16] Chaibub Neto E (2019). Detecting the impact of subject characteristics on machine learning-based diagnostic applications. Digit. Med..

[17] Hernandez-Lemus E, Vabalas A, Gowen E, Poliakoff E, Casson AJ (2019). Machine learning algorithm validation with a limited sample size. PLoS ONE.

[18] Whelan R, Garavan H (2014). When optimism hurts: inflated predictions in psychiatric neuroimaging. Biol. Psychiat..

[19] Jbabdi S (2018). Multivariate classification of neuroimaging data with nested subclasses: biased accuracy and implications for hypothesis testing. PLoS Comput. Biol..

[20] Cawley GC, Talbot NLC (2010). On over-fitting in model selection and subsequent selection bias in performance evaluation. J. Mach. Learn. Res..

[21] Torres EB, Donnellan AM (2015). Editorial for research topic “Autism: the movement perspective”. Front. Integr. Neurosci..

[22] Casartelli L, Molteni M, Ronconi L (2016). So close yet so far: motor anomalies impacting on social functioning in autism spectrum disorder. Neurosci. Biobehav. Rev..

[23] Cook JL, Blakemore S-J, Press C (2013). Atypical basic movement kinematics in autism spectrum conditions. Brain.

[24] Varlet M, Marin L, Lagarde J, Bardy BG (2011). Social postural coordination. J. Exp. Psychol. Hum. Percept. Perform..

[25] Becchio C, Sartori L, Castiello U (2010). Toward you: the social side of actions. Curr. Dir. Psychol. Sci..

[26] Trujillo JP, Simanova I, Bekkering H, Özyürek A (2018). Communicative intent modulates production and comprehension of actions and gestures: a Kinect study. Cognition.

[27] Sinha P (2014). Autism as a disorder of prediction. Proc. Natl. Acad. Sci. U S A.

[28] Mason AH, MacKenzie CL (2005). Grip forces when passing an object to a partner. Exp. Brain Res..

[29] Bates CJ, Yildirim I, Tenenbaum JB, Battaglia P (2019). Modeling human intuitions about liquid flow with particle-based simulation. PLoS Comput. Biol..

[30] Arthur T, Vine S, Brosnan M, Buckingham G (2020). Predictive sensorimotor control in autism. Brain.

[31] Woo C-W, Chang LJ, Lindquist MA, Wager TD (2017). Building better biomarkers: brain models in translational neuroimaging. Nat. Neurosci..

[32] Wechsler D (2003). Wechsler Intelligence Scale for Children.

[33] American Psychiatric Association (2013). Diagnostic and Statistical Manual of Mental Disorders.

[34] Lord C, Rutter M, DiLavore PC, Risi S, Gotham K, Bishop S (2012). Autism Diagnostic Observation Schedule, Second Edition (ADOS-2) Manual (Part I): Modules 1–4.

[35] Rutter M, Le Couteur A, Lord C (2003). The Autism Diagnostic Interview-Revised (ADI-R).

[36] Anderson P, Anderson V, Lajoie G (1996). The tower of London test: validation and standardization for pediatric populatons. Clin. Neuropsychol..

[37] Oldfield RC (1971). The assessment and analysis of handedness: the Edinburgh inventory. Neuropsychologia.

[38] World Medical, A (2013). World Medical Association Declaration of Helsinki: ethical principles for medical research involving human subjects. JAMA.

