# Supplementary material for: Identifying the signature of prospective motor control in children with autism
Source: Sci Rep. 2021 Feb 4;11:3165. doi: 10.1038/s41598-021-82374-2 (PMC7862688; doi:10.1038/s41598-021-82374-2)
Supplement: Supplementary file 1 — Supplementary Informations. [file 41598_2021_82374_MOESM1_ESM.docx]

***Supplementary Information***

**Identifying the signature of prospective motor control in children with autism**

Andrea Cavallo, Luca Romeo, Caterina Ansuini, Francesca Battaglia, Lino Nobili, Massimiliano Pontil, Stefano Panzeri and Cristina Becchio

***Supplementary Table S1.*** *Definition of kinematic parameters of interest.*

| **Kinematic parameter** | **Definition** |
| --- | --- |
| *reach onset* | time at which the wrist velocity crossed a 20 mm/s threshold and remained above it for longer than 100 ms |
| *reach offset* | the time at which the wrist velocity dropped below a 20 mm/s threshold^a^ |
| *wrist velocity* | module of the velocity of the marker on the child’s wrist (mm/sec) |
| *wrist acceleration* | first derivative of the velocity of the marker on the child’s wrist (mm/sec^2^) |
| *wrist jerk* | second derivative of the velocity of the marker on the child’s wrist (mm/sec^3^) |
| *wrist height* | z-component of the marker on the child’s wrist (mm) |
| *grip aperture* | distance between the marker placed on the tip of the thumb and the marker placed on the tip of the index finger (mm) |

^a^ For trials in which the wrist velocity did not drop below the 20 mm/s threshold, the local minimum of the velocity trace before lifting was used as reach offset. Each trajectory was visually inspected to verify the appropriateness of these criteria.


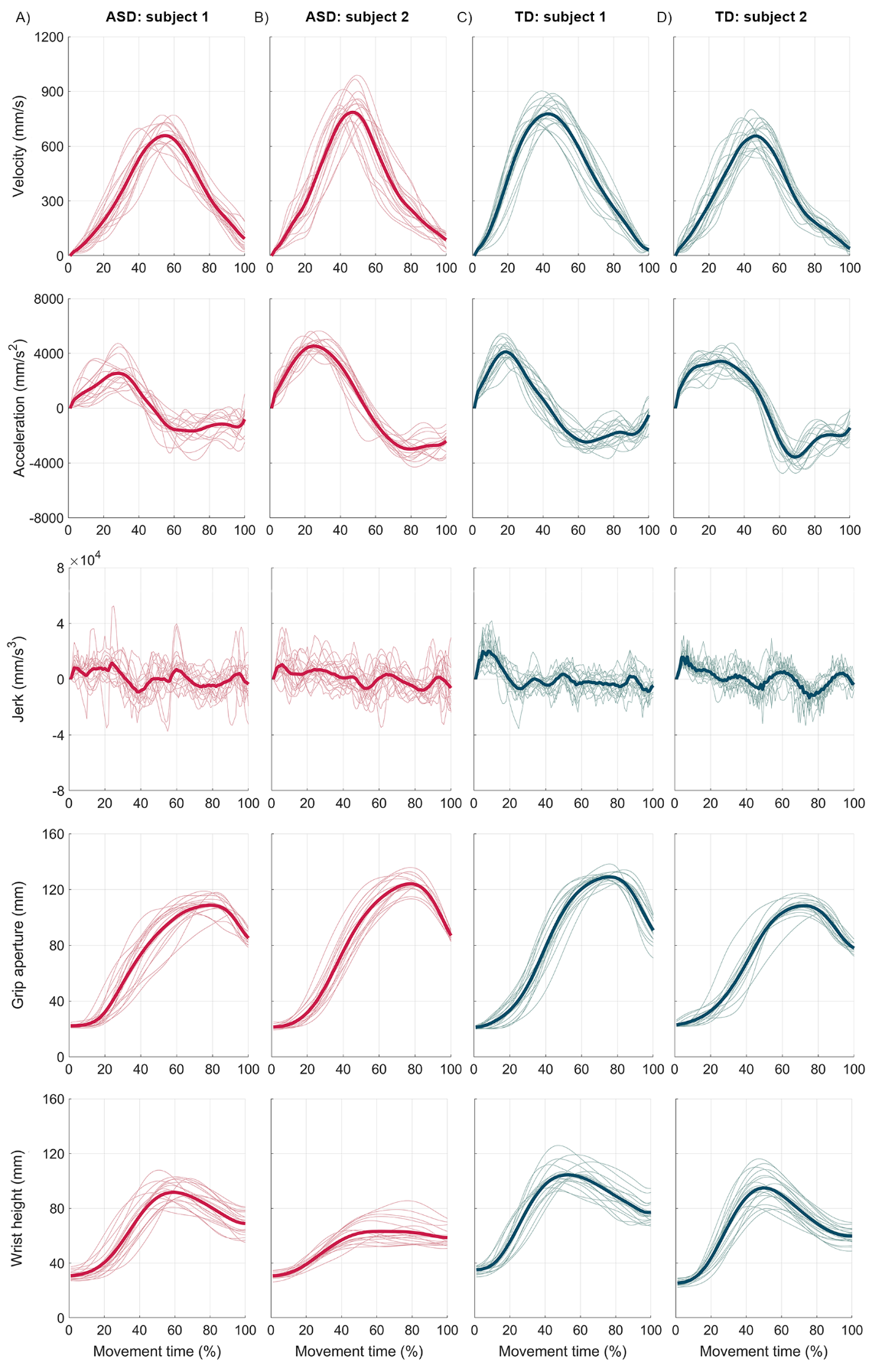


***Supplementary Figure S1.*** *Exemplar kinematic profiles of two ASD and two TD participants across 20 grasp-to-pass movements.* From top to bottom, wrist velocity, wrist acceleration, wrist jerk, grip aperture and wrist height. Thick lines represent trial average profiles of individual participants. Thin lines represent individual trials.


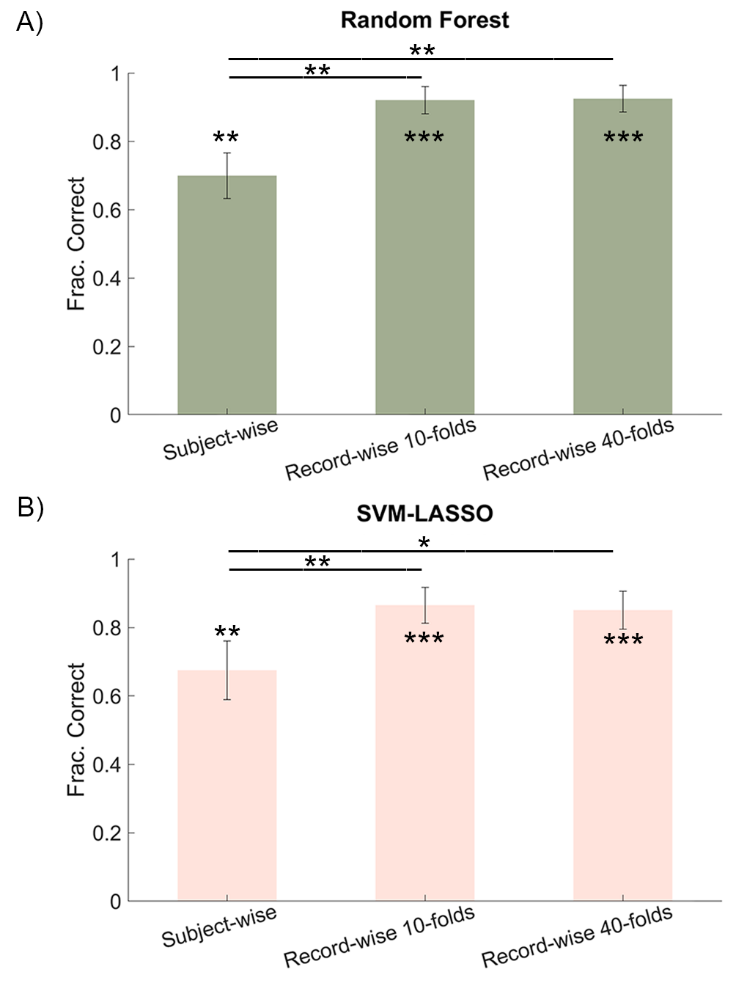


***Supplementary Figure S2.*** *Group classification accuracy (computed as fraction of correctly classified subjects) using Random Forest (A) or SVM-LASSO (B) classifiers*. We compare subject-wise, record-wise 10 folds and record-wise 40 folds cross-validation methods. Histograms represent mean ± SEM.


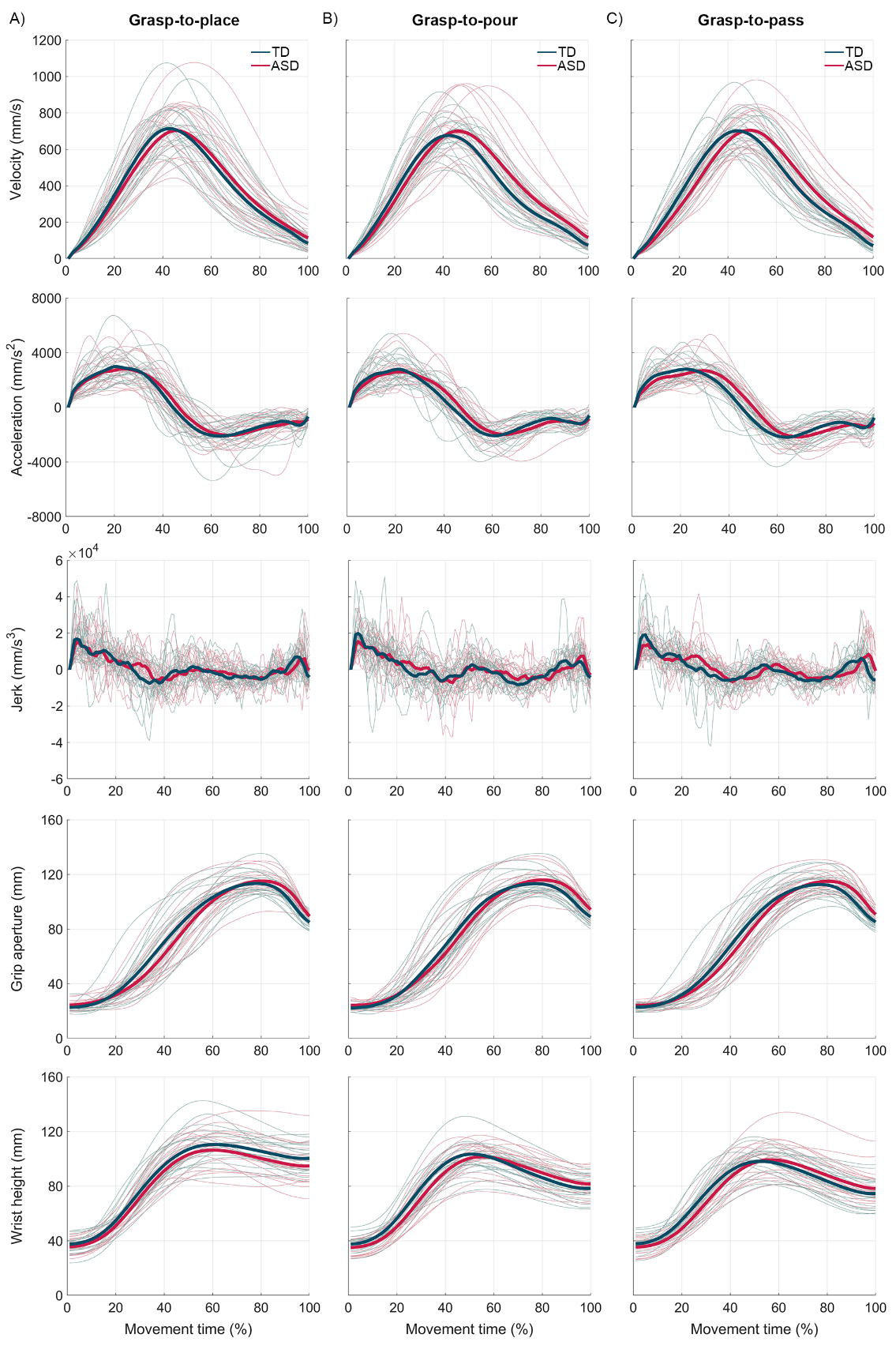


***Supplementary Figure S3.*** *Kinematic profiles of individual participants graphed by intention.* From top to bottom, wrist velocity, wrist acceleration, wrist jerk, grip aperture and wrist height. Thick lines represent group average (red = ASD group, green = TD group). Thin lines represent trial-average of individual participants.
